# Bayesian Time‐Series Models in Single Case Experimental Designs: A Tutorial for Trauma Researchers

**DOI:** 10.1002/jts.22614

**Published:** 2020-11-17

**Authors:** Prathiba Natesan Batley, Ateka A. Contractor, Stephanie V. Caldas

**Affiliations:** ^1^ Department of Life Sciences Brunel University London United Kingdom; ^2^ Department of Psychology University of North Texas Denton Texas USA

## Abstract

Single‐case experimental designs (SCEDs) involve obtaining repeated measures from one or a few participants before, during, and, sometimes, after treatment implementation. Because they are cost‐, time‐, and resource‐efficient and can provide robust causal evidence for more large‐scale research, SCEDs are gaining popularity in trauma treatment research. However, sophisticated techniques to analyze SCED data remain underutilized. Herein, we discuss the utility of SCED data for trauma research, provide recommendations for addressing challenges specific to SCED approaches, and introduce a tutorial for two Bayesian models—the Bayesian interrupted time‐series (BITS) model and the Bayesian unknown change‐point (BUCP) model—that can be used to analyze the typically small sample, autocorrelated, SCED data. Software codes are provided for the ease of guiding readers in estimating these models. Analyses of a dataset from a published article as well as a trauma‐specific simulated dataset are used to illustrate the models and demonstrate the interpretation of the results. We further discuss the implications of using such small‐sample data‐analytic techniques for SCEDs specific to trauma research.

## 抽象

簡體及繁體中文撮要由亞洲創傷心理研究學會翻譯

JOTS‐20‐0149.R2 Natesan Batley

Traditional Chinese

標題: 用於單一個案實驗設計的貝葉斯時間序列模型:為創傷研究員而設的教學

撮要: 單一個案實驗設計(SCEDs)需要在樣本接受治療前、治療期間, 及有時在他們完成治療後, 對一名或幾名參加者重複進行測量。由於SCEDs符合成本、時間及資源效益, 又能為較大型的研究提供嚴謹的因果證據, 其受越來越多創傷治療研究使用。可是, 目前仍少研究以優良技術分析SCED數據。因此, 我們探討SCED數據對創傷研究的功用, 就面對SCED方法特殊帶來的困難提供建議, 並介紹兩個貝葉斯(Bayesian)模型的教學──貝葉斯間斷時間序列設計(BITS)模型, 與貝葉斯不明改變點(BUCP) 模型;它們能用以分析小型樣本、自動關連的SCED數據。我們提供軟件編碼, 方便指導讀者對這些模型進行估算。我們分析一份已發表文獻的數據組, 及一個創傷特殊的模擬數據組, 來示範模型及對模型的結果詮釋。我們亦進一步探討以這種小樣本數據分析技術, 檢視特殊用於創傷研究的SCEDs的意味。

Simplified Chinese

标题: 用于单一个案实验设计的贝叶斯时间序列模型:为创伤研究员而设的教学

撮要: 单一个案实验设计(SCEDs)需要在样本接受治疗前、治疗期间, 及有时在他们完成治疗后, 对一名或几名参加者重复进行测量。由于SCEDs符合成本、时间及资源效益, 又能为较大型的研究提供严谨的因果证据, 其受越来越多创伤治疗研究使用。可是, 目前仍少研究以优良技术分析SCED数据。因此, 我们探讨SCED数据对创伤研究的功用, 就面对SCED方法特殊带来的困难提供建议, 并介绍两个贝叶斯(Bayesian)模型的教学──贝叶斯间断时间序列设计(BITS)模型, 与贝叶斯不明改变点(BUCP) 模型;它们能用以分析小型样本、自动关连的SCED数据。我们提供软件编码, 方便指导读者对这些模型进行估算。我们分析一份已发表文献的数据组, 及一个创伤特殊的仿真数据组, 来示范模型及对模型的结果诠释。我们亦进一步探讨以这种小样本数据分析技术, 检视特殊用于创伤研究的SCEDs的意味。

Although randomized control trials (RCTs) were once heralded as the gold standard of treatment design, researchers and policymakers have come to recognize that a one‐size‐fits‐all model is not appropriate for all research. In recent years, single‐case experimental designs (SCEDs), which permit a reasonably rigorous experimental evaluation of intervention effects and provide an avenue to examine causal effects, have become an increasingly recognized alternative to RCTs for establishing evidence of treatment efficacy and shown their utility as a method to conduct pilot investigations for large‐scale causal studies (Smith, 2012). For instance, SCEDs are appropriate to intensely pilot‐test new vaccines on small groups of individuals diagnosed with COVID‐19; their progress can be tracked across time before conducting an RCT in a larger sample. Of note, evidence from a single SCED cannot affirm causation; SCED data across both studies and researchers is needed to make strong conclusions regarding treatment efficacy (Kratochwill et al., 2010). Similarly, SCEDs are not immune to traditional methodology influences regarding the interpretation of causal relations, such as measurement and construct validity, confounding variables, and replicability and stability of obtained causal relations (Brewer, 2000). Advantageously, SCEDs are related to the goals of personalized (i.e., precision) medicine initiatives outlined in President Barack Obama's 2015 State of the Union address (Office of the Press Secretary, 2015) and are considered to be at the apex of the evidence hierarchy by the Oxford Centre for Evidence‐Based Medicine (Howick et al., 2018).

Broadly, the goal of SCEDs is to show that the observations made during a treatment phase are the function of the treatment alone (i.e., there are no alternative explanations for outcomes). A form of interrupted time‐series designs, SCEDs employ time as the independent variable, with outcome variables recorded repeatedly for individual participants. They are generally conducted in phases: a baseline phase to first establish what is typical for the participant, followed by treatment phases (Smith, 2012). Moreover, SCEDs meet the high evidence requirements set by the What Works Clearinghouse (WWC; Kratochwill et al., 2010), such as providing evidence of treatment effects immediately following introduction to and withdrawal from the treatment (i.e., the immediacy of effect) as well as evidence of consistency and stability within phases and differences in levels and/or slopes between phases. With their simplistic yet unique design, SCEDs have wide applicability to trauma research. Herein, we (a) outline the importance of SCEDs in trauma research, (b) discuss the challenges of using SCEDs, and (c) provide a description and tutorial of the Bayesian interrupted time‐series (BITS) and Bayesian unknown change‐point (BUCP) models, using an example from a published study and simulated data.

There are limitations in existing psychopathology treatment research, including posttraumatic research. First, large‐scale RCTs require moderate‐to‐large samples, take time from initiation or implementation to publication of findings (Ioannidis, 1998), and partly contribute to the concerning time lag in integrating research findings into actionable clinical practice (Morris et al., 2011). Second, psychopathology treatment research, for valid reasons, utilizes homogenous samples and excludes diagnostically complex cases (e.g., individuals with suicidal attempts; Kennedy‐Martin et al., 2015) as well as participants from diverse groups (Triffleman & Pole, 2010). However, research targeting diagnostically complex and culturally diverse or difficult‐to‐access traumatized samples is needed for evidence‐based trauma treatments and to combat the one‐size‐fits‐all assumption with regard to research and clinical practice (Steenkamp & Litz, 2013). Third, few existing methodologies permit the examination of the nuanced and cascading effects that targeting one symptom can have on other symptoms (e.g., the effects of targeting intrusion‐related symptoms of posttraumatic stress disorder [PTSD] on other PTSD symptoms). Such a precision‐medicine–based approach may be difficult to implement in regular clinical trial methodologies (Kessler et al., 2018). Finally, existing efficacious trauma treatments have considerable dropout rates (Imel et al., 2013) and lack effectiveness for all patients (Cusack et al., 2016).

As their design permits an examination of within‐ and between‐participant variability, SCEDs lend themselves particularly well to research on posttraumatic disorders, such as PTSD, wherein the occurrence of a traumatic event is etiologically related to symptomatology and in which symptoms fluctuate over time and context. Given that they are idiographic in nature, SCEDs allow for the examination of individual factors contributing to treatment response and nonresponse (Au et al., 2017). This is relevant to PTSD treatment, in which approximately 54% of treatment‐seeking individuals either do not respond to or drop out of treatment (Bradley et al., 2005). Frequent assessments, combined with a baseline phase, can aid in examining whether certain symptom changes are stable, coincide with treatment or trauma‐related triggers, or are linked with other symptoms. In addition, SCEDs can provide evidence of specificity of a posttraumatic intervention; for example, Kessler et al. (2018) demonstrated a decrease in specific intrusions that was associated with targeting those intrusions in an intervention. Rigorous methodological innovations with immense utility for trauma research and treatments (Bourla et al., 2018), such as real‐time monitoring methods, can be easily integrated into SCEDs (Bentley et al., 2019).

Supplemental Table S1 in Supplementary Materials S1 presents some examples of SCEDs used in trauma research to address various trauma‐related research issues, such as developing and/or piloting a new trauma treatment with applications for individuals who report a unique set of symptoms (e.g., complex PTSD), examining the effects of treatments on specific posttraumatic symptoms (e.g., intrusive memories), and obtaining a nuanced and time‐intensive observation of treatment effects on one trauma‐exposed individual. Broadly, SCEDs have the potential to advance trauma research. To introduce resources for implementing SCEDs in trauma research, we provide a description of the SCED design, its interpretation, and related challenges.

## Challenges to SCED Analysis

Visual analysis, one of the most commonly used SCED analyses, involves visually inspecting plotted data for consistency within treatment phases (i.e., baseline, treatment, and posttreatment) and examining differences in trends between phases (Gast & Ledford, 2014). Unfortunately, visual analyses have drawbacks. Immediacy, which is computed as the difference between the last three to five data points of the previous phase and the first three to five data‐points of the following phase, has no objective guidelines for interpretation. Although some consensus exists among expert researchers regarding rules to determine treatment effectiveness based on visual analyses (e.g., Kratochwill et al., 2010), such rules are not uniformly applied (Horner et al., 2012). Further, some treatments may not involve an immediacy effect; alternatively, general effects may not be apparent visually, despite clinical and statistical effectiveness (Meadan et al., 2016). Moreover, the presence of autocorrelation can confound the findings of visual analyses by contributing to decreased interrater reliability during visual analyses (Brossart et al., 2006) and more Type I errors (Maggin & Chafouleas, 2013). Thus, quantitative methods beyond visual analysis are needed to ascertain treatment effects for SCEDs (Maggin et al., 2011). However, common inferential statistical analyses fail with SCEDs because the time series are often too short (i.e., five to seven data points per phase), and data are autocorrelated and often presented as count or percentage estimates (Shadish & Sullivan, 2011). Most parametric analyses are inappropriate, as they assume independence of observations. Moreover, ordinary least squares (OLS) interval estimates of autocorrelations have severe undercoverage (Shadish et al., 2013). Maximum likelihood estimation of autocorrelated data is possible only with large datasets.

Recently, methodologists have begun investigating how Bayesian methods can be deployed to overcome the analytical challenges presented by SCED data. Bayesian methods are appropriate to analyze SCED data because they (a) work well with small samples (Gelman et al., 2013), (b) provide accurate interval estimates of autocorrelation (Shadish et al., 2013), (c) are flexible to model with autocorrelated small‐sample data (Kruschke, 2013; Natesan & Hedges, 2017), (d) provide reliable estimates of uncertainty (Natesan & Hedges, 2019), (e) work with count estimates for autocorrelated small sample data (Natesan Batley, Shukla Mehta, et al., 2020), and (f) produce credible intervals that can be interpreted probabilistically (Gelman et al., 2013). Natesan and Hedges (2017) proposed an analytic model—the BUCP model—to measure effect sizes for count data and extended the model to a multiple‐phase design to overcome the small‐data and autocorrelation challenges of SCEDs (Natesan Batley, Minka, et al., 2020). Natesan Batley, Minka, et al. (2020) applied the BITS model for count data, which are more common in SCEDs. The BUCP model is an extension of the BITS model (Natesan, 2019), which requires at least eight data points per phase and a standardized mean difference of 3 for a reasonably accurate estimation of the parameters (Natesan & Hedges, 2017). Whether samples are defined as “small” and “large” varies based on statistical application. However, five to eight data points per phase, common in SCEDs (Shadish & Sullivan, 2011), can still be considered a small sample scenario based on the requirement of the presence of at least three data points to discern a pattern in the regression framework. To encourage the application of these recent analytical developments to real‐world SCED data among trauma researchers, we next present a detailed overview of the BITS and BUCP models. Syntax and data files can be downloaded from Github, and a primer to Bayesian methodology is included in Supplementary Materials S2.

## BITS Model and Tutorial

**Figure 1:**
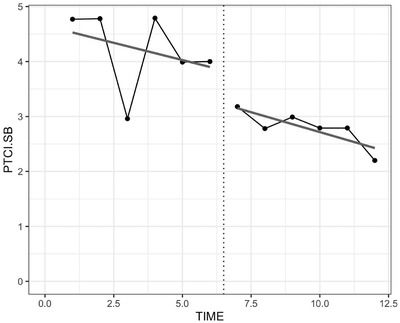
Single Case Experimental Design Plot as an Interrupted Time‐Series Design *Note*. PTCI.SB refers to Posttraumatic Cognitions Inventory Self‐Blame

The BITS model is a simple interrupted time‐series model that consists of at least two phases (i.e., baseline and treatment), with the treatment acting as the “interruption” (Figure 1). The simplest BITS model applied to SCED data consists of two phases, baseline (A) and treatment (B), and is called an “AB design.” In Phase A, the participant is observed for several time points to obtain a baseline against which treatment effects can be compared; this baseline essentially serves as the control condition. In Phase B, the treatment is implemented. The researcher fits independent lines of best fit to each phase, and, to examine treatment effectiveness, compares the intercepts, and computes the effect size as the difference between the intercepts.

In the current tutorial, we demonstrate the BITS and BUCP models using an example from a study by Au et al., (2017). In this study, a community sample of trauma‐exposed adults (*n* = 10) underwent six weekly sessions of a novel, brief compassion‐based therapy aimed to reduce trauma‐related shame and PTSD symptoms. Participants were randomly assigned to a 2‐, 4‐, or 6‐week baseline phase and completed weekly measures throughout the baseline and treatment phases. For this tutorial, we used data from the Posttraumatic Cognitions Inventory Self‐Blame subscale (PTCI‐SB; Foa et al., 1999) data for Participant P7. The PTCI (Foa et al., 1999) is a 36‐item self‐report measure that is used to assess trauma‐related patterns of thinking. Response options range from 1 (*totally disagree*) to 7 (*totally agree*). The measure yields three subscales: Negative Cognitions about the Self, Negative Cognitions about the World, and Self‐Blame. Presented data from the Au et al. (2017) study are on an interval scale.

This leads to a general discussion about the reliability and validity of SCEDs. Effect replication is a mechanism for controlling threats to internal validity and is commonly implemented in four SCED designs: multiple baseline (i.e. with at least three participants), multiphase (i.e., ABAB designs), changing criterion, and alternating treatments designs. Possible internal validity threats for SCEDs include ambiguous temporal precedence, selection bias, history‐ and maturation‐related effects, statistical regression toward the mean, attrition, testing, instrumentation, and additive or interactive effects of other threats to internal validity (Shadish et al., 2014). To address these concerns, the WWC requires that SCEDs demonstrate evidence of interrater reliability and include three demonstrations of treatment effect, a systematically manipulated independent variable, and a minimum of three data points per phase (Kratochwill et al., 2010). Evidence of an association between independent and outcome variables is established by documenting the consistency of trend, level, and variability within each phase, among other things; this serves as a rough reliability measure.

**Figure 2:**
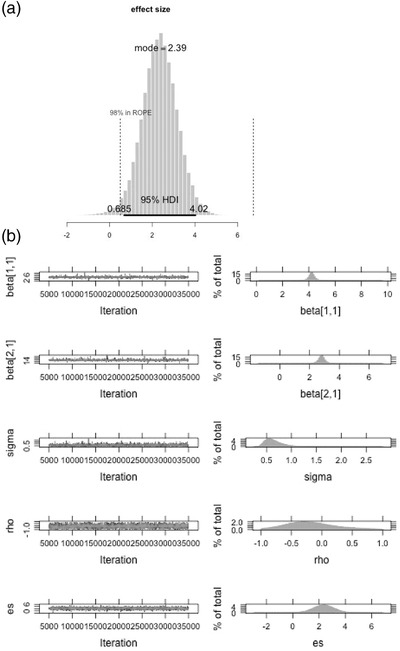
Posterior and Region of Practical Equivalence for Standardized Mean Difference Effect Size for Self‐Blame Data for Participant P7 (a) and Trace Plots and Histograms of All Estimated Parameters From the Bayesian Interrupted Time‐Series Model for Participant P7 (b)

Readers are encouraged to peruse the R script file called BITS.R as they read through this section. Before running the analyses, R (R Core Team, 2017) and JAGS (Plummer, 2003) need to be installed along with the R packages “runjags” (Denwood, 2016) and “rjags” (Plummer et al., 2019). Data for Participants P7, P8, and P9 are in a .csv file called Au_Figure2_SelfBlame.csv that is stored in the same folder as the R script file (see Supplementary Materials). The data are read using the syntax in S1:

(S1) $$\begin{array}{c} \mathrm{filename}\;<\;\;\;-\;`\mathrm{Au}\_\mathrm{Figure}2\_\mathrm{SelfBlame}`\\ \mathrm{data}\;<\;\;\;-\;\mathrm{as}.\mathrm{matrix}\left(\mathrm{read}.\mathrm{csv}\left(\mathrm{paste}0\left(\mathrm{filename},``.\mathrm{csv}"\right),\mathrm{header}=T\right)\right) \end{array}$$

We extracted data for the first participant, P7, and stored them in a 2 × 6 matrix, with “2” representing the number of rows and phases and “6” representing the number of columns and observations (Line 2, S1). Consider a continuous, normally distributed dependent variable $y_{\mathrm{pt}}$ at time point t belonging to phase p. In this case, y represents self‐blame scores and was computed as the mean of the six‐item PTCI‐SB subscale. The y values look like those shown in Table 1. Of note, the number of baseline and treatment phase observations do not have to be equal; when this occurs, the phase with fewer observations will be filled with NA (i.e., the missing data value) for the rest of the cells. The number of baseline and treatment phase observations are represented as $T_{b}$ and $T_{t}$, respectively. We can plot these data sourcing the prewritten function plot_SSD and Syntax S2. The plot is saved with the name of the participant (i.e. P7_SB‐SSDplot.jpg).

(S2) $$\begin{array}{c} \mathrm{source}\left(`\mathrm{plot}\_\mathrm{SSD}.R^{\prime }\right)\\ \;\mathrm{plot}\_\mathrm{SSD}\left(y,\mathrm{paste}0\left(\mathrm{colnames}\left(\mathrm{data}\right)\left[2\right],``-\mathrm{SSDplot}.\mathrm{jpg}"\right)\right) \end{array}$$

**Table 1:** Self‐Blame Data for Participant P7

Phase	Dependent variable					
y[1,]	4.77	4.78	2.96	4.79	3.99	4.00
y[2,]	3.18	2.78	2.99	2.79	2.79	2.20

*Note*. The first row represents baseline‐phase data. The second row represents treatment‐phase data.

### BITS Model Definition

For the model, assume that the observed value of self‐blame at $y_{p1}$ follows a normal distribution; the mean of the expected value of self‐blame at Time 1 is ${\hat{y}}_{p1}$, and the standard deviation is $\sigma_{\varepsilon }$, as shown in Equation 1 (Natesan & Hedges, 2017).

(1) $$y_{p1}\;∼\;norm\left({\hat{y}}_{p1},\sigma_{\varepsilon }^{2}\right)$$

This syntax is given in S3 for the two phases.

(S3) $$\begin{array}{c} y\left[1,1\right]∼\mathrm{dnorm}\left(\mathrm{yhat}\left[1,1\right],\mathrm{tau}\right)\\ y\left[1,2\right]∼\mathrm{dnorm}\left(\mathrm{yhat}\left[1,2\right],\mathrm{tau}\right) \end{array}$$

In JAGS, we use the dnorm function to indicate drawing from a normal distribution, and the parameters within parentheses are the mean and precision (i.e., the reciprocal of variance), respectively. Here, tau is the precision. The predicted values of self‐blame scores in the following time points, t, are given as:

(2) $$\;y_{pt}=\;{\hat{y}}_{pt}+\;\rho \left(y_{pt-1}-\;{\hat{y}}_{pt-1}\right)+\;\varepsilon$$

In Equation 2, the random error, $\varepsilon ,{\;\mathrm{has}\;a\;\mathrm{variance}\;\sigma }_{\varepsilon }^{2}$ and ρ, which is the autocorrelation between adjacent time points. This is given by Syntax S4 within a “for” loop that runs from Time 2 to $T_{b}$ (i.e., for all baseline observations).

(S4) $$y\left[1,i\right]∼\mathrm{dnorm}\left(\mathrm{yhat}\left[1,i\right]+{\mathrm{rho}}^{*}\left(y\left[1,\left(i-1\right)\right]-\mathrm{yhat}\left[1,\left(i-1\right)\right]\right),\mathrm{tau}\right)$$

In Syntax S4, rho is used to denote the autocorrelation value which is given by Equation 3. A similar syntax is applied for the treatment phase within a loop that runs from Time 2 to $T_{t}$. If e is the white noise with variance $\sigma_{e}$, it is created by a combination of autocorrelation and random error, and the relation between $\rho ,\;\sigma_{e},$ and $\sigma_{\varepsilon }$ is given in Equation 3 as:

(3) $$\;\sigma_{e}=\frac{\sigma_{\varepsilon }}{\sqrt{1-\rho^{2}}}\;$$

In SCEDs, the time series is typically expected to follow a linear procedure with lag‐1 autocorrelated errors (e.g., Natesan & Hedges, 2017). A lag‐1 autocorrelated error indicates that the error at a given time point is correlated with the error at the immediate subsequent time point and not with the error two or more time points away. The expected values of self‐blame for phase p and the serial dependency of the residual ($e_{t}$) can be expressed respectively as

(4) $$\;{\hat{y}}_{pt}=\beta_{p1}\;\;$$

(5) $$\;e_{pt}=\;\rho e_{pt-1}+\varepsilon$$

In Equation 4, $\beta_{p1}$ is the intercept of the linear regression model for phase p; $e_{\mathrm{pt}}$ is the error at time t for phase p; ε is the independently distributed error, and the value of p can be either 1 or 2, indicating the baseline and treatment phases, respectively. Thus, the syntax for Equation 4 is given as yhat[1, i] <− beta[1, 1] for all values in the baseline phase, and a similar syntax is applied for the treatment phase. The autocorrelation is traditionally set to be the same for all phases in a SCED. Effect size is defined as the standardized mean difference between the two phases, as given in Syntax S5.

(S5) $$\mathrm{es}\;<\;\;\;-\;{\left(\mathrm{beta}\left[1,1\right]-\mathrm{beta}\left[2,1\right]\right)}^{*}\mathrm{sqrt}\left(\mathrm{tau}\right)$$

Next, we define the distributions from which statistics will be drawn. In Bayesian, we specify the prior distributions (i.e., possible values parameters might take). In the BITS model, the priors are defined as follows. For the beta values, beta[i, 1] ∼ dnorm(mu[i], 1) for both phases, beta is drawn from a distribution with mean mu and precision 1, with mu differing in value for both phases. The parameter mu, in turn, is drawn from a distribution with mean 5 and precision .05 as mu[i] ∼ dnorm(5, .05). The mean value of 5 was selected because 5 is within the range of possible mean values for the PTCI‐SB subscale. To be as pessimistic as possible regarding the potential influence of the priors on the parameter estimates, we have (a) placed a prior on a prior—that is, beta is drawn from dnorm(mu, 1), and mu, in turn, is drawn from dnorm(5, .05); and (b) set the precision of the distribution to be very small, which means the variance will be 20, a rather large value that flattens the prior distribution. Thus, we minimize the influence of the prior on the parameter estimates. Readers may change the priors to evaluate whether the results vary significantly. Of note, the specification of priors on priors is called “hyperpriors” or “hierarchical priors.” Regarding tau, we draw the square root of its reciprocal—that is, the standard deviation from a less‐informative prior, as sigma ∼ dunif(0.1, 5). Again, autocorrelation is drawn from a less‐informative prior, as rho ∼ dunif(−1, 1). The simulated example in Supplementary Material S3 tests the performance of these models for three different prior specifications. Ideally, we want the estimates of $\mathrm{bet}a_{p1},\;\mathrm{rho},\;\mathrm{and}\;\mathrm{tau}$ to be independent of the prior specifications, which is what we found for the simulated dataset. This program is available as Simulation Example.R on Github.

### BITS Model Execution

For the model specified in the previous step, parallel chains with different starting values are run for many iterations until convergence is indicated. Convergence means that the parameter estimates do not fluctuate significantly. Although it is not possible to prove convergence, many statistics are used to indicate convergence. Two such convergence diagnostics were used in the present study: the multivariate potential scale reduction factor (MPSRF; Brooks & Gelman, 1998) and Heidelberger and Welch's (1983) convergence diagnostic. The package “runjags” conveniently calls the JAGS program, runs parallel chains, and iterates the model estimates until convergence is indicated. In the syntax presented later, four parallel chains are run, with starting values (i.e., random values of parameters assigned to a chain) independently generated for each chain from the prior distribution. Credible starting values can speed convergence. However, to negate the effects of starting values on the final posterior distribution, estimates from the first few thousand iterations in each chain are not included in the posterior distribution; this process is called “burn‐in” and is automatically done in the Syntax S6. Posterior distribution is the distribution that contains all possible values of the estimated parameter. In Syntax S6, the model is defined for BITS and stored in an object called BITS.model1; input data are y (i.e., observations in the form of a two‐row matrix), P (i.e., number of phases), $T_{b}$ (i.e., number of baseline observations), and $T_{t}$ (i.e., number of treatment observations). Beta, sigma, rho, and es are the parameters of interest to obtain and monitor convergence. The syntax is set to run four chains; after burning in, we obtain another 30,000 iterations per chain, on which convergence is assessed, and we provide starting values using the function called “inits.”

(S6) $$\begin{array}{ccc} & & \;\;\;\;\;\mathrm{results}\;<\;\;\;-\;\mathrm{autorun}.\mathrm{jags}(\mathrm{model}=\mathrm{BITS}.\mathrm{model}1,\\ & & \;\;\;\;\;\;\;\mathrm{data}=\mathrm{list}\left(y=y,P=P,\mathrm{Tb}=\mathrm{Tb},\mathrm{Tt}=\mathrm{Tt}\right),\\ & & \;\mathrm{monitor}=c\left(``\mathrm{beta}",``\mathrm{sigma}",``\mathrm{rho}",``\mathrm{es}"\right),n.\mathrm{chains}=4,\\ & & \;\;\mathrm{startsample}=30000,\mathrm{inits}=\mathrm{function}()\\ & & \left\{\mathrm{list}\left(\mathrm{beta}=\mathrm{rbind}\left(\mathrm{rnorm}\left(1,\mathrm{beta}1,1\right),\mathrm{rnorm}\left(1,\mathrm{beta}2,1\right)\right),\right)\right)\\ & & \;\left(\left(\mathrm{sigma}=\mathrm{runif}\left(1,0.1,5\right),\mathrm{rho}=\mathrm{runif}\left(1,-1,1\right)\right)\right\},\\ & & \;\mathrm{method}=``\mathrm{rjparallel}") \end{array}$$

Results are stored in an object called “results;” the results summary will contain the summary statistics of the posteriors of all estimated parameters. This can be checked by simply executing the “results” statement. The values can be saved in a .csv file using the following syntax.

(S7) $$\mathrm{write}.\mathrm{csv}\left(\mathrm{results}\$\mathrm{summaries},\mathrm{paste}0\left(\mathrm{filename},``-\mathrm{BITSresults}.\mathrm{csv}"\right)\right)$$

Our next goal is to check the posterior of the effect size (es), which is a simple standardized mean difference effect size. We combine the values from all four chains and all iterations to finally obtain all estimated values of es using Syntax S8.

(S8) $$\begin{array}{c} \mathrm{samples}\;<\;\;\;-\;\mathrm{combine}.\mathrm{mcmc}\left(\mathrm{results}\$\mathrm{mcmc}\right)\\ \mathrm{es}\;<\;\;\;-\;\mathrm{samples}\left[```\mathrm{es}"\right] \end{array}$$

The effect size obtained from this approach automatically incorporates small‐sample correction, unlike the approach described by Hedges et al. (2012), which uses a small‐sample correction factor. Using predefined function plots, we can now check the percentage of the values in the posterior of es that are greater than a researcher‐specified value of, say, 0.5; this is called the region of practical equivalence (ROPE; Kruschke, 2013). In this case, 98% of the effect size estimates are above 0.5, which means that of the 120,000 obtained effect size estimates, 98% of them were above 0.5. Syntax S9 plots the posterior distribution and the essential details of ROPE.

(S9) $$\begin{array}{c} \mathrm{plots}(\mathrm{es},\mathrm{compVal}=1,\mathrm{ropeRad}=0.5,\mathrm{maintitle}=``\mathrm{effect}\mathrm{size}",\\ \mathrm{HDImass}=.95,\mathrm{plotname}=\mathrm{paste}0\left(\mathrm{colnames}\left(\mathrm{data}\right)\left[2\right],``-\mathrm{effect}\mathrm{size}\mathrm{rope}"\right)) \end{array}$$

In this syntax, compVal – ropeRad is the lowest value of effect size that the researcher would accept as having a practically significant treatment effect. The plot is saved under the file name P7_SB‐effect size rope.jpg (Figure 2, Panel A). Of note, if we compare the 95% credible interval of es [0.65, 4.00] to the 95% confidence interval [0.66, 1.96] computed by Au et al. (2017), we find that the interval width for the latter is smaller. This could be in line with the possible undercoverage of estimates found by Shadish et al. (2013). Alternatively, the wider credible interval for the Bayesian estimate could also be due to the relatively noninformative priors specified in the syntax. Now, we can obtain and examine visual plots of the posteriors of other parameters and their convergence using Syntax S10.

(S10) $$\mathrm{plot}\left(\mathrm{results},\mathrm{layout}=c\left(5,2\right),\mathrm{plot}.\mathrm{type}=c\left(``\mathrm{trace}",``\mathrm{histogram}"\right)\right)$$

Syntax S10 plots the trace plot, which contains information about all the estimates for a given parameter at a given iteration, and a histogram of these estimates (i.e., posterior distribution; Figure 2, Panel b). The posterior estimates indicate that the estimated mean values of the baseline and treatment phases are close to the observed mean of these phases, respectively. The autocorrelation posterior distribution is quite widely spread and includes 0, which means that whether these data are autocorrelated remains questionable. Readers are encouraged to run the syntax, which has been automated for multiple participants under the file name BITS‐looped.R.

### BITS Model Interpretation

Although the estimates obtained from the BITS model and the change score model Au et al. (2017) could not be directly compared due to differences in the models, broadly, a difference was observed in the magnitude of the treatment effect across both methods. Treatment was found to have a substantial effect on self‐blame for Participant P7 with the BITS model versus Au et al.’s (2017) change score method. Specifically, the BITS model revealed a statistically significant treatment effect greater than 0.65 (i.e., the lower CI limit), with the posterior mean estimated to be 2.33 (*SD* = 0.8), 95% CI [0.65, 4.00], representing a medium‐to‐large effect size. In contrast, according to the change‐score model, the treatment effect was not statistically different from 0 (i.e., 95% CI [0.00, 3.60], contained 0). These differences could be attributed to autocorrelations in the change‐score method, which are accounted for in the BITS model. In the context of the study by Au et al. (2017), the results indicated that their developed 6‐week self‐compassion intervention was effective for this participant in reducing posttraumatic self‐blame cognitions. Thus, for this specific individual, with their unique sociocultural and psychiatric history and via reduced self‐blame in the context of their experienced traumatic event, the examined self‐compassion intervention may have promise in reducing overall PTSD symptom severity.

In addition, Au et al. (2017) found an overall effect size of 1.31, 95% CI [0.66, 1.96], for all participants, based on a method described by Shadish et al. (2014) that considers autocorrelations and implements a small‐sample correction. Thus, although the treatment was not effective for Participant P7, it was effective overall. Again, we cannot compare the effect size from the BITS model, which produces individual effect size estimates, to this overall effect size for all participants. We note here that the Shadish et al. (2014) effect size does not consider trends in the data (i.e., growth or declining patterns), but the BITS model can accommodate such model specifications.

## BUCP Model and Tutorial

The BUCP model, an extension of the BITS model, can be used to model interrupted time‐series SCED data with only a few autocorrelated data points. The presented BUCP model is a confirmatory approach that allows the data to speak for themselves. In a two‐phase design, it assumes that the change point (i.e., the point at which the treatment is introduced) is unknown. If the algorithm estimates the change point accurately with a narrow credible interval (i.e., the Bayesian equivalent of a confidence interval), there is sufficient confirmatory evidence of both immediacy and treatment effects.

### BUCP Model Definition and Execution

The program is given in the R script titled BUCP.R. The intercept $\beta_{p1}$ can be modeled as:

(6) $$\beta_{p1}=\;\beta_{11}dummy+\;\beta_{21}\left(1-dummy\right)$$

where

(7) $$\mathrm{dummy}\;=\;\left\{\begin{array}{c} 1,\;\;\mathrm{if}\;t\leq t_{b}\\ 0,\;\;\mathrm{otherwise} \end{array}\right.\;\;$$

Earlier, we used two “for” loops, one each for the baseline and treatment phases. However, to use a for loop in most programs, the user needs to know the bounds of the loop a priori (e.g., 1 to 10). However, popular Bayesian packages such as JAGS and BUGS (Lunn et al., 2009) use the function called “step” to circumvent this problem. We create two dummy variables, one called “dummy” that is used to indicate the phase, using the Syntax S11.

(S11) $$\mathrm{dummy}\left[i\right]<\;\;\;-\;\mathrm{step}\left(\mathrm{CP}-i\right)$$

This means that if the given time point i is less than the change point CP, step will assign a value of 1 to dummy and 0 otherwise. Based on this, another dummy variable called “temp” is used to compute the mean of the distribution from which the dependent variable (i.e., self‐blame) needs to be drawn. This is given in Syntax S12

(S12) $$\mathrm{temp}\left[i\right]<\;\;\;-\;\mathrm{dummy}\left[i\right]{}^{*}\mathrm{beta}\left[1,1\right]+\left(1-\mathrm{dummy}\left[i\right]\right){}^{*}\mathrm{beta}\left[2,1\right]$$

When the dummy is 1, the data will belong to the baseline phase, and the second half of the right‐hand side of this syntax will vanish as it is being multiplied by 1‐dummy (i.e., 0). When the dummy is 0, the data will belong to the treatment phase, as the first half of the right‐hand side of this syntax will vanish, as it is being multiplied by 0.

**Figure 3:**
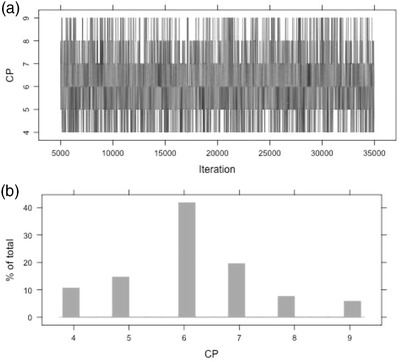
Trace Plot (a) and Histogram (b) of Change Point (CP) for Self‐Blame for Participant P7

The only other new parameter in this model is the change point, which is estimated as a categorical variable with integer values ranging from 4 to (T – 3). These bounds are specified because we need at least three observations to create a pattern in which the change point cannot happen during the first three time points or the last three time points of the experiment. The posterior of the change point in Figure 3 indicates that although values from 4 to 9 are all possible values, Time 6 is the most probable value for the change point. This shows support for the treatment effect. The top panel of Figure 3 shows part of the trace plot for the change point. Because the graph remains in the same region from 5,000 to 35,000 iterations, the estimates possibly converged to stationarity. Alternatively, we consider the posterior means for the baseline and treatment phases for the BITS and BUCP models which were similar in value; the results indicate that Time 6 is a good estimate of the change point. As with BITS, the program called BUCP‐looped.R runs these analyses for multiple participants. Summaries of posterior distributions of Participant P7's self‐blame data from BITS and BUCP models are given in Table 2.

**Table 2:** Summaries of Posteriors of Bayesian Interrupted Time Series and Bayesian Unknown Change‐Point Models

	95% CI				
Parameter	Lower limit	Median	Upper limit	*M*	*SD*
BITS model					
beta[1,1]	3.60	4.22	4.91	4.24	0.33
beta[2,1]	2.17	2.81	3.49	2.82	0.33
sigma	0.35	0.62	1.05	0.66	0.20
rho	−0.96	−0.21	0.65	−0.17	0.41
es	0.65	2.34	4.00	2.33	0.85
BUCP model					
CP	4.00	6.00	9.00	6.17	1.25
beta[1,1]	3.15	4.06	4.82	4.03	0.42
beta[2,1]	1.98	2.84	3.70	2.84	0.43
sigma	0.40	0.72	1.24	0.77	0.24
rho	−0.22	0.12	0.51	0.13	0.18
es	0.89	2.63	4.20	2.65	0.83

*Note*. BITS = Bayesian interrupted time series; BUCP = Bayesian unknown change point; es = effect size, CP = change point.

### BUCP Model Interpretation

The BUCP model results show that although values between 4 and 9 are probable candidates (i.e., estimates) for the changepoint, 6 is the most commonly estimated change‐point value. Because 6 is also the true time point the intervention was introduced, there is medium evidence of immediacy effect, a required piece of evidence of an intervention effect. The effect sizes of the BITS and BUCP models were comparable. Although the data pattern in the study by Au et al. (2017) provided a medium level of evidence of immediate impacts of the self‐compassion intervention on posttraumatic self‐blame, the effect size between the two phases was medium to large, supporting further consideration for professionals interested in this self‐compassion intervention. Au et al. (2017) did not compute immediacy. When immediacy was computed as the difference between the medians of the last three observations of the baseline phase and the first three observations of the treatment phase, we obtained a value of 1.01. However, as mentioned, there is no rule of thumb for how to interpret the magnitude of this statistic, unlike the change point, whose posterior shape and 95% credible interval indicate the strength of immediacy.

In the simulated example (Supplementary Material S3), all estimated values were close to the true values for both the BITS and the BUCP models regardless of the prior specification. The change point was estimated accurately, showing support for immediacy. The data showed support for a treatment effect, as the posterior mean of the effect size was between 6.17 and 6.59 for all models and priors, which is considered a large standardized mean difference effect size (Harrington & Velicer, 2015).

## Discussion

Herein, we have presented an overview, description, and tutorial of the BITS and BUCP models to evaluate trauma‐specific treatment data published by Au et al. (2017) as well as trauma‐specific simulated data. We included detailed syntax associated with these models in R (available on Github) to equip trauma researchers with cutting‐edge statistical tools with which to analyze SCED data. Trauma researchers and clinicians would benefit from understanding and capitalizing on the value of SCEDs to pilot new posttrauma treatments or modify or augment an existing treatment to aid diverse individuals presenting with complex or low base‐rate symptomatology. If used in conjunction with robust statistical approaches such as BITS and BUCP models, SCED data can reduce costs toward treatment research and implementation by funneling effort and resources toward trauma treatments that estimate the magnitude as well as the immediacy of effects in pilot studies. In fact, the immediacy of treatment effects may be more pertinent to trauma‐exposed individuals because of the higher levels of functional impairment seen in this population (Norman et al., 2007). Further, Bayesian analyses, although computationally intensive, work for small‐sample data as generated by SCED in a time‐effective manner. We encourage educational programs to impart training in Bayesian approaches to enhance competencies in this technique; Bayesian approaches may be seen as a good alternative to frequentist methods by gatekeepers of research, such as funding agencies and journal editors (Natesan Batley, Boedeker, et al., 2020). Finally, SCEDs are a clinically valid method for identifying the differences in efficacy between treatment form or dosage with a small number of participants.

This said, we highlight a few caveats to this statistical approach. First, there is a learning curve associated with Bayesian methods; this can be overcome with more training, editorial policies, and tutorial papers, such as the present study. Second, the programs are not a “point‐and‐click” solution like those some researchers may be used to. Third, SCEDs may be burdensome for researchers who want to implement treatment immediately, as they tend to have a shorter time series, and these Bayesian models require at least eight data points per phase. Fourth, as stated in the WWC standards (Kratochwill et al., 2010), confidence in the evidence of treatment effects produced by SCEDs is enhanced by the replication of effects across different studies, cases, and research groups (Horner et al., 2012). Thus, the evidence from a single study, such as that demonstrated in this paper, must be interpreted with caution. Fifth, SCEDs do not concern themselves with lasting treatment effects, even after the treatment is removed. Thus, the persistence of these effects is not tested in the models currently in use for SCED analyses. However, future research should consider the incorporation of posttreatment follow‐up and the long‐term effects in SCEDs, as this would be a more sustainable and clinically relevant model. Finally, some considerations specific to trauma research may interact with these aforementioned caveats; these include high dropout rates in trauma treatment studies (Imel et al., 2013), the impact of high‐level of distress in trauma‐exposed individuals regarding their ability or willingness to wait to start treatment while participating in the baseline phase (Stecker et al., 2013), and fluctuations in posttraumatic symptoms on a daily (i.e., short‐term) basis (Chun, 2016), rendering it difficult to get a stable baseline estimate before implementing trauma treatment. In conclusion, the ability of SCEDs to examine individual factors related to the development or nondevelopment of pathology following trauma exposure and treatment outcomes and remission provides tremendous value for preventive and remedial trauma intervention research.

## Open Practices Statement

We analyzed archival data that are not under our direct control but that were already published and available to the public. We also simulated data for the illustration dataset in the Supplemental Materials. Our complete analysis data, scripts, and codes can be freely downloaded and modified for researchers’ personal use from Github (https://github.com/prathiba-stat/BITS-BUCP). Thus, both data and scripts are freely available for public use.

### Open Research Badges

This article has been awarded Open Data and Open Materials badges. All data and materials are publicly accessible at http://www.iris-database.org.

## Supporting information

Supporting Material
