# Supplementary material for: Bayesian Time‐Series Models in Single Case Experimental Designs: A Tutorial for Trauma Researchers
Source: J Trauma Stress. 2020 Nov 17;33(6):1144–53. doi: 10.1002/jts.22614 (PMC8246830; doi:10.1002/jts.22614)

**Supplementary Material S1**

**Table 1A**

*Detailed information for studies using variants of single case experimental designs in trauma research.*

| **Study Aims** | **Design** | **Measurement** | **Analyses** | **Findings** |
| --- | --- | --- | --- | --- |
| Au et al., 2017; Community sample of trauma-exposed adults (*N* = 10) | | | | |
| Develop brief compassion-based therapy & assess efficacy for reducing trauma-related shame & PTSD symptoms | Multiple baseline design (randomized & nonconcurrent) | -Pre-Tx phase: Weekly; 2-, 4-, or 6-week period  -Tx phase: Weekly; 6-weeks  -FU phase: At 2- & 4-weeks | -Review of W/in & B/w outcome data plotted graphically  -W/in change scores  -Comparison of W/in & B/w Tx phase level & slope of outcome compared to baseline  -Sdiff & *d*-statistic*  -Correlations of change scores  -Examination of retention rates, treatment credibility, satisfaction ratings, & qualitative feedback  -Therapist observations | - Following Tx, 9 of 10 participants demonstrated reliable decreases in PTSD severity & shame, maintained at 2- & 4-week FU  -Tx associated with improved self-compassion & reduced self-blame |
| Cowles et al., 2017; Adolescent female (*N* = 1) with depression, social anxiety, panic, & CFS | | | | |
| Detail recognizing & assessing PTSD in a previously undiagnosed adolescent, & using individualized TF-CBT for PTSD from multiple traumas | AB single-case quasi-experimental design | -Pre-Tx: At 10 months, 1 month, & 1 week pre-Tx  -Tx phase: Weekly; 15 sessions  -FU phase: At 3 months | -Review of outcome data plotted graphically  -Descriptive statistics  -T-scores for depression/anxiety (mother & patient)  -Weekly symptom/impact referencing PTSD scores  -Therapist observations | -Following Tx, PTSD, panic, social anxiety, & depression reduced from clinical to non-clinical levels, maintained at 3-month FU |
| DuHamel et al., 2000­­; 40-year-old man BMT with PTSD (*N* = 1) | | | | |
| Investigate utility of specific trauma-focused intervention (BMT-TFI) for PTSD symptoms  in BMT survivors | Single case design | -Pre-Tx: At baseline  -Tx phase: Clinical observation during 10-week intervention  -FU phase: At 1-week, & 1-, 6-, & 12-months | -Critical difference test based on *z* scores with retest reliability coefficient  -Therapist observations | -No longer met criteria for PTSD post-Tx, results maintained at 6-months FU  -Intervention associated with reduced distress & PTSD symptoms, & improved quality of life |
| Feather et al., 2006; Children who have experienced multiple traumas with PTSD (*N* = 4) | | | | |
| Pilot manualized TF-CBT | Multiple baseline across subjects with between-person replications (randomized & non-concurrent) | -Pre-Tx: Weekly; 3-, 5-, 7-, or 9-week period  -Tx Phase: Weekly; 16 weeks  -FU phase: At 3-, 6-, & 12-month | -Inspection of baseline data to determine stability  - Review of W/in & B/w outcome data plotted graphically  -Descriptive statistics  -Mean change scores for self-report measures  -Therapist observations | -Following Tx, PTSD symptoms decreased; effects maintained at 3-, 6-, & 12-month FU  -Tx associated with improvements in self-perceived coping ability post-Tx & over 12-month FU period |
| Kessler et al., (2018); Patients with complex PTSD in inpatient Tx (*N* = 20) | | | | |
| Test novel intervention to reduce frequency of intrusive memories among inpatients with complex PTSD | Multiple baseline design (not randomized) | -Pre-Tx: 3x/day across ~1-2 weeks  -Tx phase: 3x/day & weekly; ~3-8 weeks  -FU phase: 3x/day ~1-2 weeks | -Percentage reduction in mean symptom frequency  -Repeated measures ANCOVA (B/w)  -Paired sample *t* test (W/in)  -Linear regression with two-tailed Mann–Whitney *U* to test reduction in slopes of “intervals of interest” & all other intervals.  -Comparison of change in outcome scores  -Pearson correlations of change scores  -Therapist observations | -Following Tx, frequency of targeted intrusions decreased by average of 64% (vs. 11% for non-targeted intrusions)  -80% of participants experienced >50% reduction in intrusion frequency |

*Note*. Tx = treatment; FU = follow-up; W/in = within-subject; B/w = between-subject; TFI = Trauma-Focused Intervention; TF-CBT = trauma-focused cognitive behavioral therapy; CFS = chronic fatigue syndrome; BMT = Bone marrow transplantation; SDiff = Standard error of the difference; *d*-statistic = standardized mean difference effect size; ANCOVA = analysis of covariance; PTSD = posttraumatic stress disorder; * = specifically for single-case design.

**References**

Au, T. M., Sauer-Zavala, S., King, M. W., Petrocchi, N., Barlow, D. H., & Litz, B. T. (2017). Compassion-based therapy for trauma-related shame and posttraumatic stress: Initial evaluation using a multiple baseline design. *Behavior Therapy, 48*(2), 207-221. <https://doi.org/10.1016/j.beth.2016.11.012>

Cowles, M., & Davis, J. (2017). Identifying, assessing and treating complicated post-traumatic stress disorder in adolescence: A single-case quasi-experimental design with clinical case report. *The Cognitive Behaviour Therapist, 10*. <https://doi.org/10.1017/S1754470X17000010>

DuHamel, K. N., Ostroff, J. S., Bovbjerg, D. H., Pfeffer, M., Morasco, B. J., Papadopoulos, E., & Redd, W. H. (2000). Trauma-focused intervention after bone marrow transplantation: A case study. *Behavior Therapy, 31*(1), 175-186. <https://doi.org/10.1016/S0005-7894(00)80010-6>

Feather, J. S., & Ronan, K. R. (2006). Trauma-Focused Cognitive-Behavioural Therapy for abused children with posttraumatic stress disorder: A pilot study. *New Zealand Journal of Psychology, 35*(3), 132-145.

Kessler, H., Holmes, E. A., Blackwell, S. E., Schmidt, A. C., Schweer, J. M., Bücker, A., Herpertz, S., Axmacher, N., & Kehyayan, A. (2018). Reducing intrusive memories of trauma using a visuospatial interference intervention with inpatients with posttraumatic stress disorder (PTSD). *Journal of Consulting and Clinical Psychology, 86*(12), 1076-1090. <https://doi.org/10.1037/ccp0000340>

**Supplementary Material S2 Bayesian Methodology**

**Description and Application to Single Case Experimental Designs (SCED)**

To yield information about model parameters, Bayesian methodology uses information from (1) the data in the form of likelihood and (2) the researcher’s estimates of posterior distributions of parameters in the form of priors (prior knowledge/information on a subject). In other words, Bayesian estimation uses prior information to update the posterior, which are a set of all possible values of the parameter estimate. The general relationship between the likelihood (i.e., information from the data), the prior information about the parameters, and the posterior distribution of the parameters is given by the proportionality,

$$Posterior \propto Prior \times Likelihood$$

One of the main advantages of Bayesian estimation is the use of posterior distributions. Imagine that, instead of a point estimate and its standard error for a parameter (produced from frequentist, non-Bayesian approaches), one were able to obtain a distribution for a parameter, which not only gives us the most likely estimate in the form of the mode, but also the range of plausible values. This equips the researcher with more information on how best to trust the estimates. For instance, if one has a normal distribution with a very narrow standard deviation, one would have more confidence in using the mean of the distribution as a good representation of the parameter estimate. However, if one had a flat, uniform distribution, then the mean is as good as any value in the distribution to represent the parameter, as seen in Figure 1B.

Assume that the normal curve in Figure 1B is the Bayesian posterior distribution of an effect size. This gives us richer information than a simple point estimate, with a line segment extended on both sides, representing a confidence interval produced by frequentist approaches more commonly used in trauma research (shown in Figure 1B). Although the 95% credible interval (which is the Bayesian equivalent of confidence interval) in this case is -0.95 to 2.96, from the posterior distribution we know that values between 0 and 2 are more probable, even within the range of the credible interval than other values in this interval. Such a statement cannot be made about parameter estimates in the frequentist framework. In fact, the only information we know from the frequentist confidence interval (the horizontal line) in Figure 1B is that any value between 0 and 2 is probable; there is no probability value associated with this quantification.

If we go a step further, we can interpret the 95% credible interval as the probability of the true value being contained in this interval as 0.95 (Gelman, et al., 2013). With a frequentist confidence interval interpretation, the 95% confidence interval tells us that in the presence of a hypothetical infinite number of trials, 95% of those confidence intervals so obtained would contain the true value. The bounds of the confidence interval are statistics dependent on the sample dataset. In fact, we do not know anything about the probability of the current confidence interval containing the true value. In sum, (a) credibility intervals, are more straightforward to interpret (Gill, 2014), (b) one can accept the hypothesis in Bayesian statistical significance testing (Kruschke, 2013), and (c) SCED Bayesian effect sizes do not need to be corrected for small sample size.

Especially for complex models, Bayesian estimation has been shown to work well with small sample data because it does not depend on asymptotic or large sample theory (Ansari & Jedidi, 2000; Ansari et al., 2000; Fox, 2010; Kieftenbeld & Natesan, 2012, Natesan & Hedges, 2017; Natesan Batley et al., 2020). This is of particular advantage to small sample designs such as SCEDs. Commonly used maximum likelihood-based estimates require large sample sizes in order to produce asymptotically unbiased estimates, which is a problem for SCED data. Although Bayesian methods cannot wash away the uncertainty or magically produce better estimates, it allows the use of prior information, which can reduce this uncertainty in the estimates (e.g. Natesan & Hedges, 2017; Natesan Batley et al., 2020; Rindskopf, 2014; Shadish et al., 2013). In addition, Bayesian methods can accommodate count and proportion data that are more common in SCEDs. Finally, Bayesian methods offer the flexibility to accommodate model complexities such as autocorrelations. In fact, Bayesian credible intervals of autocorrelations in SCEDs have been shown to be more accurate than frequentist confidence intervals (Shadish et al., 2013).

**Bayesian Priors**

Inappropriate priors lead to inappropriate posteriors, especially in small samples. Obviously for small sample data or for data that contain relatively little information, the impact of the priors on the posterior will be large. Therefore, priors should be chosen carefully, especially in small sample cases such as SCEDs. Relatively uninformative or hyperpriors can have minimal influence on the estimates (e.g. Natesan et al., 2016). On the other hand, researchers can obtain meaningful priors from results of previous published studies or through informed decision-making about the qualities of the parameters or the research design; such meaningful priors can yield better posterior estimates.

The use of priors should not be a deterrent to statistical analysis, rather an incentive. Natesan et al. (2019) encourage the use of priors as a fully Bayesian meta-generative thinking approach that enables us to (a) acknowledge that our data are a sample that are almost never random; by including priors we systematically integrate the results from many other similar samples; and (b) situate our research into the larger body of research, not just in the literature review and data collection, but also in data analysis. Especially in small sample cases such as this, researchers might also benefit from conducting a sensitivity (to priors) analysis by testing the difference in the posterior distributions for various prior distributions. Readers may reference other valuable resources to know more about Bayesian analyses (Gelman et al., 2013; Kruschke, 2013; Lynch, 2007).

**Figure 1B**

*
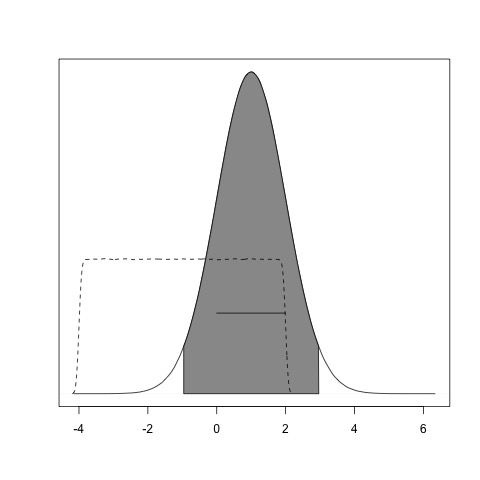
Posterior distributions of normally and uniformly distributed parameters*

*Note*. Dashed line represents a uniform distribution where the mean is as good as any estimate. Horizontal line is a frequentist confidence interval ranging from 0 to 2 with no shape to this interval. Shaded region is the 95% credible interval of effect size [-0.95, 2.96] with an interpretable shape.

**References**

Ansari, A. & Jedidi, K. (2000). Bayesian factor analysis for multilevel binary observations. *Psychometrika, 65*(2), 475-497. <https://doi.org/10.1007/BF02296339>

Ansari, A., Jedidi, K., & Jagpal, S. (2000). A hierarchical Bayesian approach for modeling heterogeneity in structural equation models. *Marketing Science*, *19*(4), 328-347*.* <https://doi.org/10.1287/mksc.19.4.328.11789>

Fox, J-P. (2010). *Bayesian item response modeling: Theory and applications*. Springer.

Gelman, A., Carlin, J. B., Stern, H. S., Dunson, D. B., Vehtari, A., & Rubin, D. B. (2013). *Bayesian data analysis* (3rd ed.). Chapman & Hall.

Gill, J. (2014). *Bayesian methods: A social and behavioral sciences approach* (3rd ed.). Chapman & Hall/CRC Press.

Kieftenbeld, V., & Natesan, P. (2012). Recovery of graded response model parameters: A Comparison of marginal maximum likelihood and Markov chain Monte Carlo estimation. *Applied Psychological Measurement, 36*(5), 399-419. <https://doi.org/10.1177/0146621612446170>

Kruschke, J. K. (2013). Bayesian estimation supersedes the t-test. *Journal of Experimental Psychology: General, 142*, 573-603. <https://doi.org/10.1037/a0029146>

Natesan, P. & Hedges, L. V. (2017). Bayesian unknown change-point models to investigate immediacy in single case designs. *Psychological Methods, 22*(4)*,* 743-759*.* <https://doi.org/10.1037/met0000134>

Natesan, P., Nandakumar, R., Minka, T., & Rubright, J. (2016). Bayesian Prior Choice in IRT estimation using MCMC and Variational Bayes. *Frontiers in Psychology: Quantitative Psychology and Measurement, 7*, 1-11. <https://doi.org/10.3389/fpsyg.2016.01422>

Natesan Batley, P., Minka, T., & Hedges, L. V. (2020). Investigating immediacy in multiple phase-change single case experimental designs using a Bayesian unknown change-points model. *Behavioral Research Methods.* <https://doi.org/10.3758/s13428-020-01345-z>

Rindskopf, D. (2014). Nonlinear Bayesian analysis for single case designs. *Journal of School Psychology, 52*(2), 179-189. <https://doi.org/10.1016/j.jsp.2013.12.003>

Shadish, W. R., Rindskopf, D. M., Hegdes, L. V., & Sullivan, K. J. (2013). Bayesian estimates of autocorrelations in single-case designs. *Behavioral Research Methods, 45*(3)*,* 813-821. <https://doi.org/10.3758/s13428-012-0282-1>

**Supplementary Material S3 Simulation Example**

We generated data for a hypothetical study that measured PTSD symptom severity on a scale of 0 to 80 with the PTSD Checklist for DSM-5 (PCL-5; Weathers et al., 2013). The aim of this hypothetical study was to examine if a certain implemented trauma treatment (i.e., Prolonged Exposure; Foa et al., 2019) resulted in reduced PTSD severity for one participant based on change in PCL-5 scores in baseline (A) and treatment (B) phase. Consider data that are plotted from a normal distribution with baseline mean of 60 and a treatment phase mean of 50, each with standard deviation of 1 (these are the true population values). We assumed the autocorrelation to be 0.2 and we assumed to have 8 observations in each phase; 8 sessions were chosen based on the minimum of 8 sessions required in Prolonged Exposure (Foa, 2011). We generated data using the R package gsarima (Briet, 2015); these data are plotted in Figure 1C.

To illustrate both the BUCP model and the impact of priors on the estimates, we examined the BUCP model with different priors. Table 1C provides detailed results, and the traceplots and histograms of the change-points are given in Figures 2C, 3C, and 4C for all three priors. First, the BUCP model was used with priors (prior set A) for the means of the baseline and treatment phases obtained from a normal distribution with a mean of 50 and a standard deviation of 1. Upon close inspection, we see that the means of the baseline (58.97) and treatment (50.30) phases were close to the population values. All parameters except the standard deviation, and therefore the effect size, were close to the true values. The standard deviation was larger than the true value, and therefore the effect size (6.29) was smaller than the true value (10).

Next, the same BUCP model was fitted to the same data, except this time with priors (prior set B) for the standard deviations of the baseline and treatment phases fixed at 1. Upon close inspection, we see that the means of the baseline (58.85) and treatment (50.30) phases were close to the population values as for the previous set of priors. Again, the standard deviation was higher (1.4) and therefore the effect size (6.14) lower than the original values.

Last, a third set of priors (prior set C) was used where the standard deviation of the dependent variable was drawn from a gamma distribution with the shape and rate fixed at 1 each. Again, all parameters were close to the true values except the standard deviation (1.30) and effect size (6.54). Inspection of the traceplots and histograms of the change-points (Figures 2C, 3C, and 4C) for all three priors indicated that parameters were close to the true value most of the time. Thus, for this simulated data, the estimates were not substantially affected by prior choice.

Irrespective of the prior specification, the change-point was always correctly estimated to be at 8 with almost no fluctuation as seen by its 95% CIs. This shows strong evidence of immediacy. The posterior mean of the effect size was estimated to be between 6.17 to 6.59 for the different prior specifications. If the trauma researcher hypothesizes that a treatment that produces a standardized mean difference of at least 3 would be considered significant, the research hypothesis that the treatment was effective would be accepted (in the Bayesian framework, see Kruschke, 2013). This is because the lower limit of the 95% CI is above 3 for all prior specifications.

**References**

Briet, O. (2015). Package “gsarima”. Retrieved from https://cran.r-project.org/web/packages/gsarima/gsarima.pdf.

Foa, E. B. (2011). Prolonged exposure therapy: Past, present, and future. *Depression and Anxiety*, *28*(12), 1043-1047. <https://doi.org/10.1002/da.20907>

Foa, E. B., Hembree, E. A., Rothbaum, B. O., & Rauch, S. A. M. (2019). *Prolonged Exposure Therapy for PTSD: Emotional processing of traumatic experiences.* Oxford University Press.

Weathers, F. W., Litz, B. T., Keane, T. M., Palmieri, P. A., Marx, B. P., & Schnurr, P. P. (2013). *The PTSD Checklist for DSM-5 (PCL-5)*. <https://www.ptsd.va.gov/professional/assessment/adult-sr/ptsd-checklist.asp>

**Table 1C**

*Results of Bayesian unknown change-point (BUCP) models with different prior sets.*

|  | Prior Set A | |  | Prior Set B | |  | Prior Set C | |
| --- | --- | --- | --- | --- | --- | --- | --- | --- |
| Parameter | Mean | SD |  | Mean | SD |  | Mean | SD |
| CP | 8.03 | 0.17 |  | 8.04 | 0.20 |  | 8.02 | 0.15 |
|  | [8, 8] |  |  | [8, 8] |  |  | [8, 8] |  |
| beta[1,1] | 58.94 | 0.60 |  | 58.80 | 0.63 |  | 58.86 | 0.56 |
|  | [57.7, 60.07] |  |  | [57.52, 59.97] |  |  | [57.74, 59.96] |  |
| beta[2,1] | 50.30 | 0.54 |  | 50.29 | 0.55 |  | 50.29 | 0.50 |
|  | [49.18, 51.34] |  |  | [49.2, 51.36] |  |  | [49.29, 51.28] |  |
| sigma | 1.44 | 0.34 |  | 1.46 | 0.36 |  | 1.36 | 0.29 |
|  | [0.89, 2.12] |  |  | [0.89, 2.16] |  |  | [0.86, 1.92] |  |
| rho | 0.02 | 0.03 |  | 0.02 | 0.03 |  | 0.02 | 0.03 |
|  | [-0.04, 0.08] |  |  | [-0.03, 0.08] |  |  | [-0.03, 0.07] |  |
| es | 6.29 | 1.49 |  | 6.17 | 1.51 |  | 6.59 | 1.46 |
|  | [3.48, 9.29] |  |  | [3.28, 9.17] |  |  | [3.78, 9.49] |  |

*Note.* SD = standard deviation; es = effect size; CP = change-point; rho = autocorrelation; 95% credible intervals are given in brackets.

**Figure 1C**

*Simulated data showing PTSD symptom severity during baseline and treatment phases*


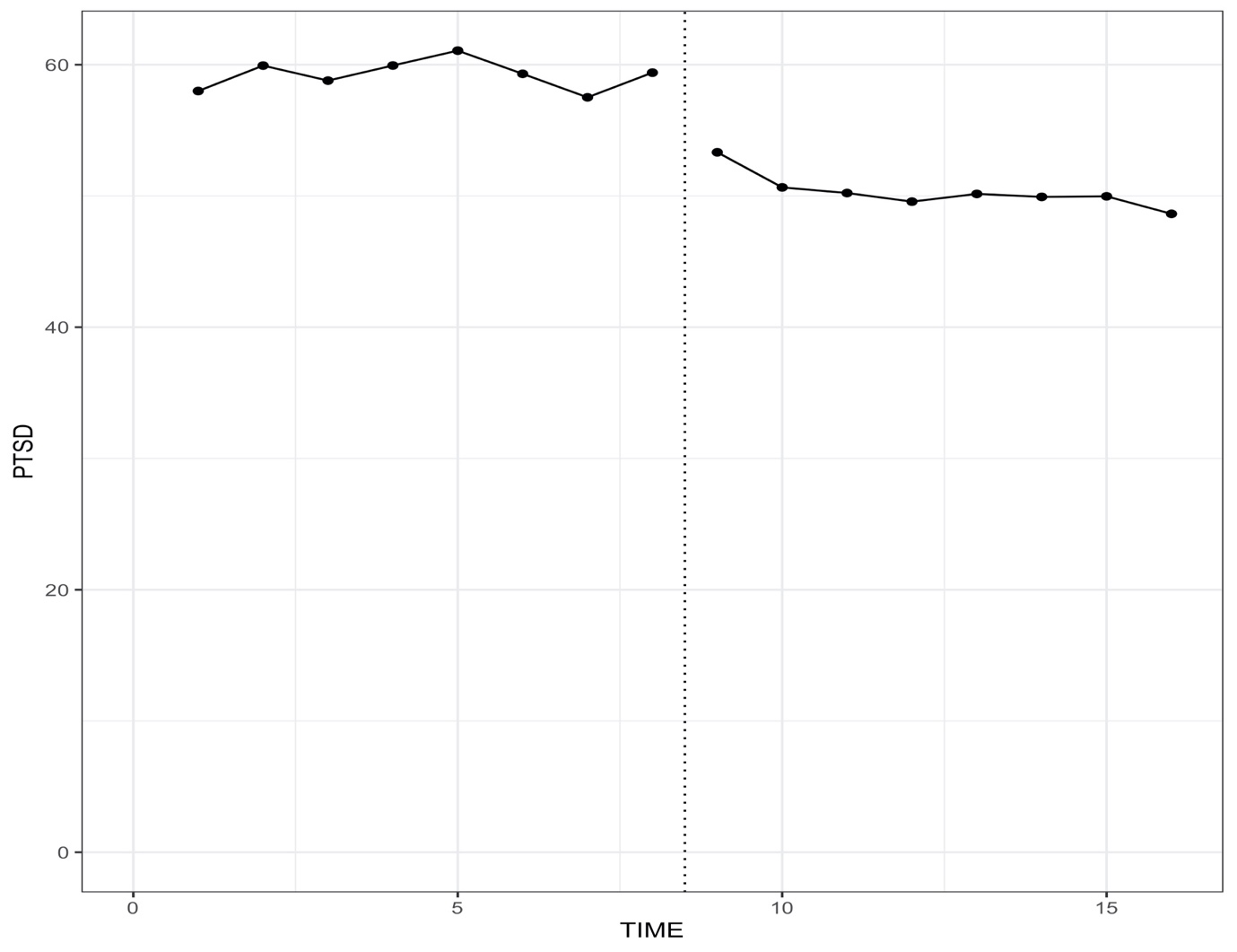


**Figure 2C**

*Traceplot and histogram of change-point (CP) from prior set A*


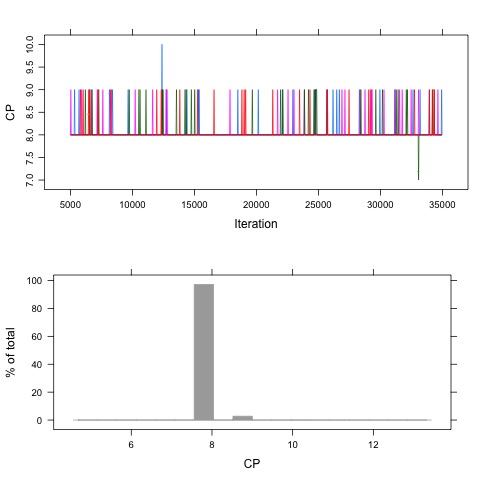


**Figure 3C**

*Traceplot and histogram of change-point (CP) from prior set B*


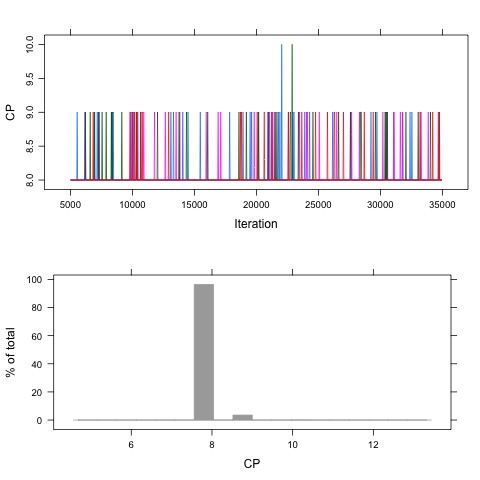


**Figure 4C**

*Traceplot and histogram of change-point (CP) from prior set C*


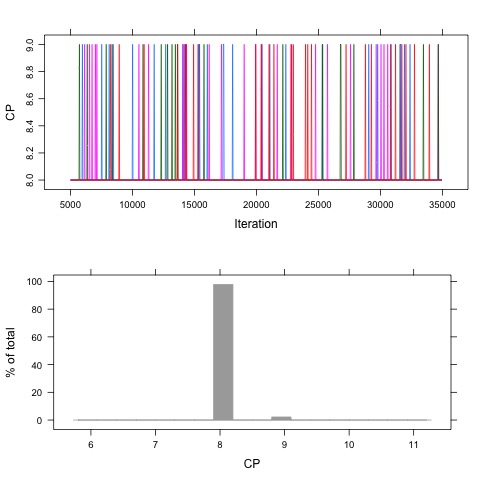

Supplement: Supplementary file 1 — Supporting Material [file JTS-33-1144-s001.docx]
